# Glucagon like receptor 1/ glucagon dual agonist acutely enhanced hepatic lipid clearance and suppressed *de novo* lipogenesis in mice

**DOI:** 10.1371/journal.pone.0186586

**Published:** 2017-10-24

**Authors:** Vijay R. More, Julie Lao, David G. McLaren, Anne-Marie Cumiskey, Beth Ann Murphy, Ying Chen, Stephen Previs, Steven Stout, Rajesh Patel, Santhosh Satapati, Wenyu Li, Edward Kowalik, Daphne Szeto, Andrea Nawrocki, Alessandro Pocai, Liangsu Wang, Paul Carrington

**Affiliations:** 1 Cardiometabolic Diseases Biology- Discovery, Merck & Co., Inc., Kenilworth, NJ, United States of America; 2 In Vitro Pharmacology, Merck & Co., Inc., Kenilworth, NJ, United States of America; 3 In-Vivo Pharmacology, Merck & Co., Inc., Kenilworth, NJ, United States of America; East Tennessee State University, UNITED STATES

## Abstract

Lipid lowering properties of glucagon have been reported. Blocking glucagon signaling leads to rise in plasma LDL levels. Here, we demonstrate the lipid lowering effects of acute dosing with Glp1r/Gcgr dual agonist (DualAG). All the experiments were performed in 25 week-old male diet-induced (60% kCal fat) obese mice. After 2 hrs of fasting, mice were injected subcutaneously with vehicle, liraglutide (25nmol/kg) and DualAG (25nmol/kg). *De novo* cholesterol and palmitate synthesis was measured by deuterium incorporation method using D_2_O. ^13^C_18_-oleate infusion was used for measuring fatty acid esterification. Simultaneous activation of Glp1r and Gcgr resulted in decrease in plasma triglyceride and cholesterol levels. DualAG enhanced hepatic LDLr protein levels, along with causing decrease in content of plasma ApoB48 and ApoB100. VLDL secretion, *de novo* palmitate synthesis and fatty acid esterification decreased with acute DualAG treatment. On the other hand, ketone levels were elevated with DualAG treatment, indicating increased fatty acid oxidation. Lipid relevant changes were absent in liraglutide treated group. In an acute treatment, DualAG demonstrated significant impact on lipid homeostasis, specifically on hepatic uptake, VLDL secretion and *de novo* synthesis. These effects collectively reveal that lipid lowering abilities of DualAG are primarily through glucagon signaling and are liver centric.

## Introduction

According to International Diabetes Federation (IDF), in 2012 worldwide there were 415 million people diagnosed with diabetes mellitus, and if significant measures are not taken, this epidemic is expected to affect 642 million people by the year 2040. In United States alone, 44.3 million patients were diagnosed with diabetes in 2015, which is 14% of the total population [1]. Type 2 diabetes (T2D) is more commonly occurring type, and it is often associated with obesity. T2D is characterized by hyperglycemia, abnormally elevated glucagon secretion, mild to moderate hyperinsulinemia and dyslipidemia.

A collaborative meta-analysis of 102 prospective studies revealed that diabetes increases risk of vascular disease at least twofold [2]. This translates primarily to diabetic dyslipidemia, as intensive glycemic control was unable to significantly reduce coronary heart disease risk [3]. Diabetic dyslipidemia primarily features high triglyceride (TG) levels, and reduction in high-density lipoprotein (HDL) cholesterol. Insulin resistance leads to accumulation of VLDL, partially through VLDL secretion defects- as reviewed by [4].

The complex nature of diabetic disorder necessitates use of combination therapies that will not only reduce hyperglycemia, but also correct other elements of diabetes complication, such as dyslipidemia. An increasingly common approach in peptide therapeutics focuses on hybrid peptides that can target two or even three receptors. Oxyntomodulin, a peptide secreted from enteroendocrine L-cells demonstrated weak binding and activation on both glucagon-like peptide 1 receptor (Glp1r) and glucagon receptor (Gcgr) [5]. Similar dual agonist peptides, with improved half-life and potency have been reported [6]. In comparison to Glp1r agonist alone, Glp1r/ Gcgr dual agonist (DualAG) exhibited superior glycemic control and improvemed dyslipidemia [6].

Following chronic treatment, the long-acting DualAG not only induced greater weight loss, but also improvements in plasma leptin, insulin, adiponectin were more pronounced compared to Glp1r agonist. Rates of fatty acid oxidation, as measured by betahydroxybutyrate, were noted to increase with DualAG treatment [6]. It has been known for several decades that glucagon signaling can effect whole body metabolism beyond glycemia, for example pharmacological injection of glucagon in rats increases metabolic rate and reduces body weight [7]. However, the role of glucagon signaling has been under-appreciated in the context of whole body lipid homeostasis. Recent rodent and clinical studies with glucagon receptor antagonists (GRAs) have shown that impairment of glucagon signaling increases plasma LDL-cholesterol [8]. Further, that the absence of glucagon receptor made mice resistant to diet-induced weight gain and hepatic steatosis [9].

The chronic effects of dual Glp1r/Gcgr agonism in rodents are well established, and include reductions in body weight, hepatic steatosis, fat mass and circulating lipids [6]. To elucidate the direct mechanistic impact of glucagon agonism on lipids in the context of GLP1R/GCGR coagonism on lipid parameters and to isolate these from effects secondary to reductions in food intake and body weight, compared the lipid lowering effects of DualAG, compared to Glp1r agonist liraglutide (Lira) immediately following dosing. The mechanism for altered lipid homeostasis by dual agonism of Glp1r and Gcgr is illustrated.

## Materials and methods

### Animals

All the experiments were performed in male C57BL/6 mice, fed standard rodent chow or high-fat diet (Diet-induced obese, DIO, D12492; 60% kcal from fat; Research Diets). The animals were single housed, and maintained on 12hr light/ 12hr dark cycle. Animal procedures used in following experiments were approved by the research laboratories of Merck & Co., Inc., Kenilworth, NJ USA, Institutional Animal Care and Use Committee.

### Peptide

The peptide was synthesized by standard Solid-phase Peptide Synthesis (SPPS) using Fmoc/t-Bu chemistry. The assembly was performed on a Rink-amide PEG-PS resin, Champion (Biosearch Technologies, 0.28 mmol/g) on a Symphony (Protein Technologies) peptide synthesizer. All the amino acids were dissolved at a 0.3 M concentration in DMF. The amino acids were activated with equimolar amounts of HATU (O-(7-azabenzotriazol-1-yl)-N,N,N',N'-tetramethyluronium hexafluorophosphate) solution 0.3 M in DMF, and a 2-fold molar excess of DIEA (N,N-diisopropylethylamine), solution 2M in NMP. The acylation reactions were performed in general for 1 hour with a 5-fold excess of activated amino acid over the resin free amino groups with double 45minutes acylation reactions performed from His1 to Thr7 and from F22 toV23.

The side chain protecting groups were: tert-butyl for Glu, Ser, D-Ser, Thr and Tyr; trityl for Asn, Gln and His; tert-butoxy-carbonyl for Lys, Trp; and, 2,2,4,6,7-pentamethyldihydrobenzofuran-5-sulfonyl for Arg; His was introduced as Boc-His(Trt)-OH at the end of the sequence assembly. At the end of the synthesis, the dry peptide-resins were individually treated with 25 mL of the cleavage mixture, 88% TFA, 5% phenol, 2% triisopropylsilane and 5% water for 1.5 hours at room temperature. Each resin was filtered and then added to cold methyl-t-butyl ether in order to precipitate the peptide. After centrifugation, the peptide pellets were washed with fresh cold methyl-t-butyl ether to remove the organic scavengers. The process was repeated twice. Final pellets were dried, resuspended in H2O, 20% acetonitrile, and lyophilized. The crude peptides (140 mg in 3 ml of DMSO) were purified by reverse-phase HPLC using preparative Waters Deltapak C4 (40 x 200 mm, 15 μm, 300Ǻ) and using as eluents (A) 0.1% TFA in water and (B) 0.1% TFA in acetonitrile. Analytical HPLC was performed on a Acquity UPLC Waters Chromatograph with a BEH300 C4 Acquity Waters column 2.1x100 mm, 1.7μm, at 45°C, using H2O, 0.1% TFA (A) and CH3CN, 0.1% TFA (B) as solvents. The peptides were characterized by electrospray mass spectrometry on an Acquity SQ Detector.

### Glp1r and Gcgr potency of the peptide

Cisbio cyclicAMP assay was used in order to determine half maximum effective concentration of the peptide necessary for activation of Glp1r and Gcgr. The peptide was diluted in assay buffer and incubated with Chinese hamster ovary cells stably transfected with murine Glp1r and Gcgr. After adding Cisbio detection reagents, the cAMP was detected by decrease in time-resolved fluorescence energy transfer (TR-FRET) using an EnVision platereader (PerkinElmer).

### Single time-point acute dosing study

DIO mice (n = 6/ group) were fasted for two hours prior to dosing vehicle (5mM sodium phosphate saline, pH 8, with 5% mannitol, dosed at the volume of 5ml/kg), 25nmol/kg Liraglutide or 25nmol/kg DualAG subcutaneously (s.c.). Blood and tissues were collected after 6 hrs of dosing, while animals still fasting. Blood glucose levels for this and all other experiments were measured from tail bleeds with glucometer (One Touch Ultra, Lifescan Inc).

### Multiple time-point acute dosing study

DIO mice (n = 4/ group per time point) were fasted for two hours prior to dosing vehicle or 25nmol/kg DualAG s.c. Blood and tissues were collected at 0.5hr, 1hr, 2hrs, 3hrs and 6hrs post dosing.

### RNA isolation and quantitative RT-PCR

Total RNA was isolated from liver tissue by SV RNA Isolation System (Promega), and quantified by Nanodrop. Followed by reverse transcription, the cDNA was subjected to quantitative RT-PCR using the ABI Prism 7700 Sequence Detection System (Applied Biosystems). Taqman probes were purchased from Thermo Fisher Scientific. Expression of beta-actin was used to normalize the gene expression.

### Protein extraction and Western blot

Total protein extracts were made from liver tissue by RIPA buffer containing protease inhibitor and phosphatase inhibitor tablets (Roche). By using BCA assay, proteins were quantified. About 20microgram protein was resolved on SDS-PAGE gels, transblotted on PVDF membrane and incubated with antibodies specific for LDLr, Pcsk9, Abca1, Abcg1, Abcg5 or tubulin. The signal was detected by Odyssey Imaging System (LI-COR Biosciences). Blots were quantified by ImageJ.

### Hepatic *de novo* lipogenesis assay

To quantify acute *de novo* cholesterol and fatty acid synthesis, deuterated water tracer method was used as described previously [10]. DIO mice were fasted for 2 hrs, injected with 20ml/kg ^2^H_2_O (Sigma-Aldrich) along with vehicle, DualAG and Liraglutide treatment. After 6 hrs of treatment, blood was collected to isolate plasma. Plasma (30μl) was spiked with C17:0 internal standard heptadecanoic acid and then saponified with 1N potassium hydroxide in 80% ethanol. Samples were acidified with 6N hydrochloric acid and lipids were extracted with chloroform. Following evaporation of the solvent, the residue was first reacted with trimethylsilyldiazomethane to form fatty acyl methyl esters, evaporated to dryness and then reacted with pyridine-acetic anhydride (1:1 v/v) at 65°C for 30 mins to generate cholesterol acetate. The reagents were evaporated to dryness. The 2H labeling of the derivatized lipids was determined using Agilent 5973-MSD equipped with an Agilent 6890 GC system, a DB5-MS capillary column (30m*0.25mm*0.25mm). The mass spectrometer was operated in the electron impact module, using selective ion monitoring of m/z 270 and 271 for palmitate, 284 and 285 for C17:0, and 368 and 369 for cholesterol; data were collected using selected ion monitoring at a dwell time of 10ms per ion. To quantify contribution of lipid synthesis, a precursor product labeling ratio was assumed and the general equation was used:

%newlymadematerial=productlabeling/(precursorlabeling*n)*100)

for which product labeling = palmitate or cholesterol; precursor: body water; n = number of exchangeable hydrogens.

For *de novo* lipogenesis assay from tissues, 100mg liver tissue was homogenized in PBS, and 100μl of the homogenate was used in place of 30μl plasma, and rest of the procedure was followed as described above. The data obtained was normalized to tissue weight.

### Monoglyceride acyltransferase (MGAT) and diglyceride acyltransferase (DGAT) activity assay

Enzymatic activity assay for MGAT and DGAT were performed on liver tissue microsomal extract from mice dosed with vehicle, Liraglutide or DualAG. Mgat activity was measured by quantifying incorporation of ^14^C-oleoyl moiety into diacylglycerol using ^14^C-oleol CoA donor and monoacylglycerol acceptors. Dgat activity was measured by incorporation of ^14^C-oleoyl moiety into trioleoylglycerol. Methods were described in detail previously [11]. Recombinant human Mgat2 and Dgat1 were used as a positive control for validating the assay.

### Hepatic TG metabolism measurement using ^13^C_18_-oleate

Dynamic kinetics of hepatic TG metabolism was determined using stable isotope tracer platform as described previously [12]. Three hours prior to the stable isotope injection, male DIO mice (n = 8/group) were injected vehicle, DualAG or Lira s.c. Two hours after treatment injection, mice were given DGAT2 inhibitor or microsomal TG transfer protein inhibitor (MTPi) by oral gavage in the control groups. All the groups received intravenous 50mg/kg ^13^C_18_-oleate three hours after vehicle, DualAG or Lira dosing. The blood was collected 5, 10, 20 and 30 minutes post tracer injection to analyze for newly synthesized TG by tracer enrichment, as described previously [12].

### VLDL secretion study

DIO mice were fasted overnight, followed by baseline blood collection (20μl). Mice were injected with vehicle, Lira or DualAG s.c. at the doses described above. After one hour of treatment, the mice were injected with non-ionic detergent Poloxamer-407 (1g/kg i.p.). Blood was collected from mice at 1, 2, 4 and 6 hr post Poloxamer dosing. TG was quantified from the plasma by biochemical analysis.

### Biochemical analysis of lipids, insulin, ketones and PCSK9

Following commercial kits were used to analyze biochemical parameters from mouse plasma: Insulin (ELISA, Alpco), βHBA (LiquiColor, Stanbio Laboratories), and TG (LiquiColor Mono, Stanbio Laboratories). Pcsk9 was measured using in-house ELISA (Merck & Co., Inc., Kenilworth, NJ USA), the protocol was described previously [13].

### Statistics

All the data is presented as mean ± SD, unless specified otherwise. One-way ANOVA was performed for comparing treatment groups to control, and p ≤ 0.05 (*), p ≤ 0.01 (**), p ≤ 0.001 (***) was reported statistically significant difference.

## Results

### DualAG demonstrated marginally superior efficacy over liraglutide for glucose lowering

**Fig 1:**
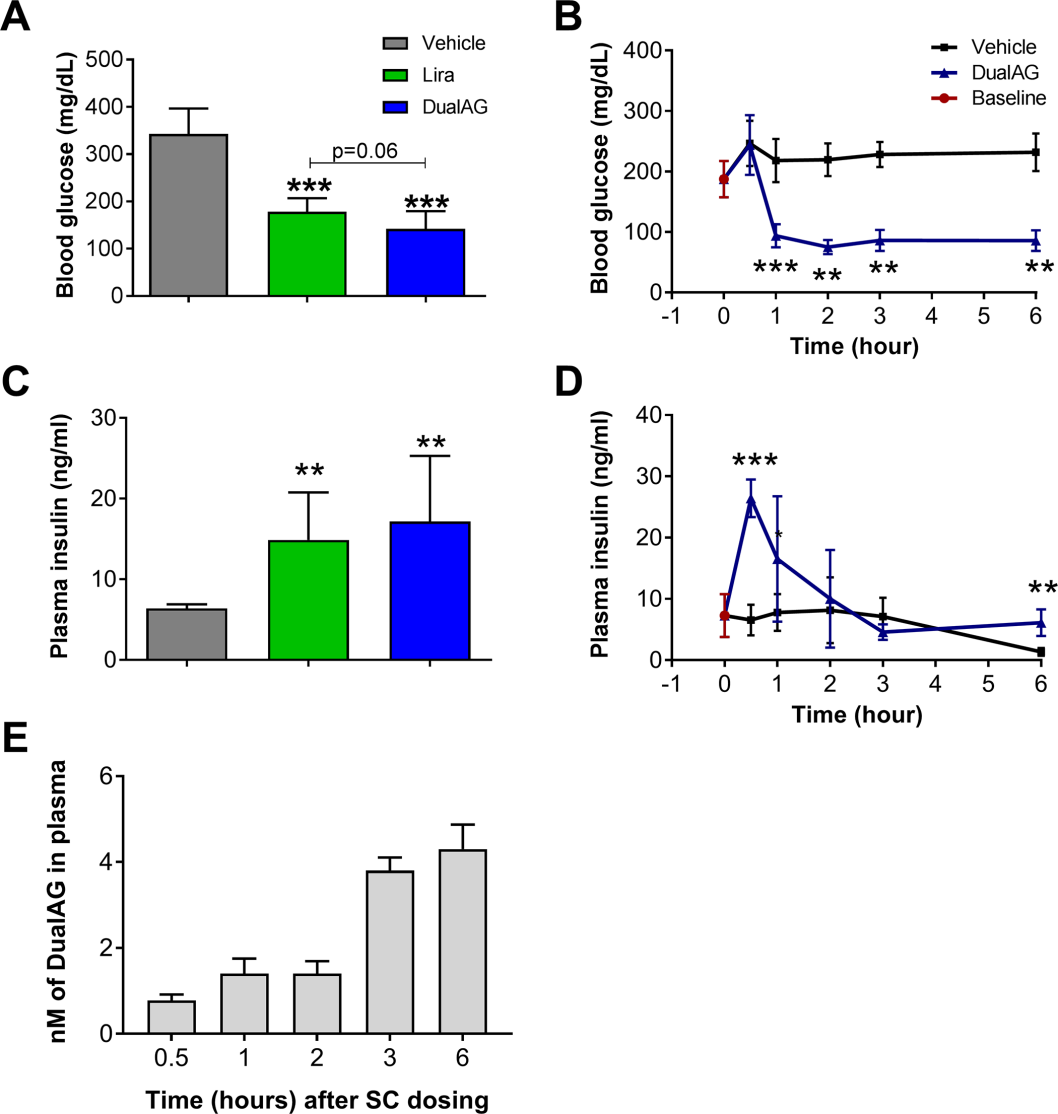
Glp1/Gcgr dual agonist (DualAG) improved glucose and insulin levels in DIO mice. Mice were fasted for 2hrs followed by injection of vehicle, Liraglutide (25nmol/kg) or DualAG (25nmol/kg). **A.** Blood glucose levels after 6 hrs of treatment injection. **B.** DualAG induced blood glucose level decrease over the course of 6 hrs after injection. **C.** Plasma insulin levels after 6 hrs of treatment injection. **D.** Plasma insulin levels monitored at multiple time-points for vehicle and DualAG treated groups. **E.** Bioavailability of the DualAG peptide during the course of the study.

In agreement with previously published studies, activation of both Glp-1 and Gcgr signaling improved blood glucose slightly more effectively compared to liraglutide alone [6]. In an acute study, 6 hrs post-injection, DualAG reduced blood glucose from 293±22 to 116±12 mg/dl. The difference between Liraglutide and DualAG blood glucose levels was not statistical (Fig 1A). Within one hour of subcutaneous injection of DualAG, blood glucose was noted to decrease and remained stable for duration of the experiment (Fig 1B). Improvement in plasma insulin levels was noted for both peptides, DualAG with a slightly higher trend compared to liraglutide (Fig 1C). DualAG induced elevation of insulin levels appeared as early as 30 minutes; however a sharp decline was noted subsequently (Fig 1D). The observed changes in blood glucose levels matched with pharmacokinetic findings, which revealed that DualAG was detected in serum at 30 minutes, with levels remaining consistently elevated for at least 6 hrs (Fig 1E).

### DualAG decreases plasma triglyceride (TG) and cholesterol

**Fig 2:**
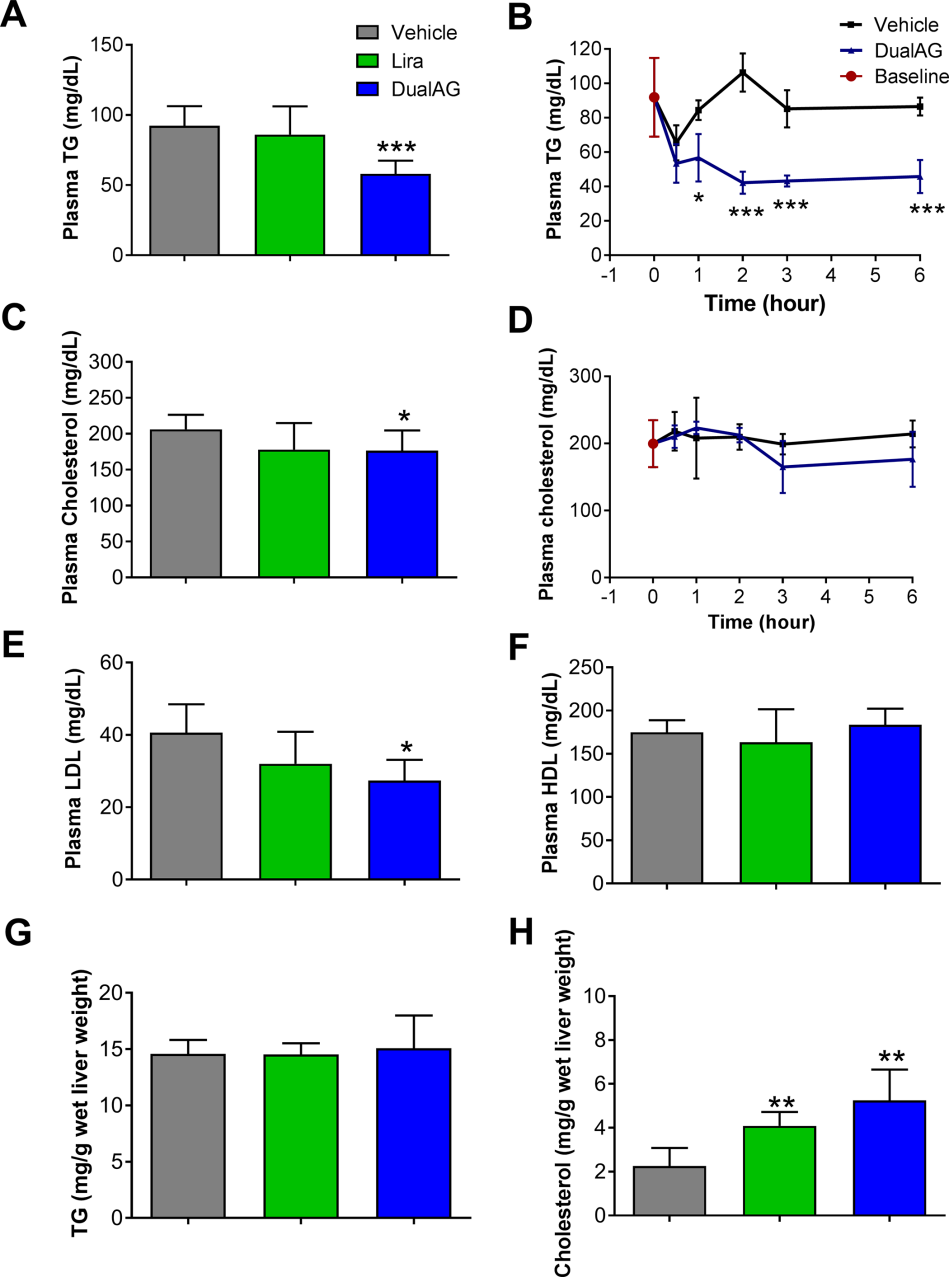
Plasma and hepatic lipids in DIO mice treated with DualAG. Mice were fasted for 2hrs followed by injection of vehicle, Liraglutide (25nmol/kg) or DualAG (25nmol/kg). **A.** Plasma TG after 6 hrs of treatment injection. **B.** Reduction in plasma TG levels by DualAG over the course of 6 hrs. **C.** Plasma total cholesterol levels at 6 hrs. **D.** Plasma cholesterol levels over the course of 6 hrs. **E.** Plasma LDL after 6 hrs of treatment injection. **F.** Plasma HDL after 6 hrs of treatment injection. **G.** TG levels in liver tissue after 6 hrs of treatment injection. **H.** Total cholesterol levels in liver tissue after 6 hrs of treatment injection.

In an acute study, DualAG treatment lowered plasma TG levels significantly, at 1, 2, 3 and 6 hrs after dosing (Fig 2A and 2B). Consistent lowering of plasma TG appeared primarily through Gcgr activation, as levels were unaffected in liraglutide treatment group. The decrease in plasma total cholesterol level was less prominent, albeit significant in DualAG treated mice. Liraglutide showed a decreased but statistically insignificant trend in total cholesterol levels (Fig 2C and 2D). Low-density lipoprotein (LDL) levels in plasma demonstrated a pattern similar to total cholesterol (Fig 2E). High-density lipoprotein (HDL)-cholesterol fraction remained unchanged with both Liraglutide and DualAG treated groups (Fig 2F).

### DualAG induced cholesterol accumulation in liver

In order to achieve a more comprehensive understanding of cholesterol and TG homeostasis alterations with DualAG treatment, liver tissue was extracted with methanol-chloroform, and lipids were analyzed by biochemical assay. Hepatic TG levels remained unchanged with both liraglutide and DualAG acute treatment (Fig 2G). Total cholesterol levels in liver increased 2-fold with liraglutide and 5-fold with DualAG compared to vehicle (Fig 2H). This increase in hepatic accumulation of cholesterol could be attributed to a combination of one or more of these three factors; first, higher uptake of peripheral cholesterol by liver, second through enhanced de-novo synthesis in liver, and third by suppressed secretion of cholesterol from liver to plasma. All of these three mechanisms were tested in acute treatment of DualAG, and are explained in the following sections.

### DualAG increases hepatic cholesterol/ TG uptake relevant targets

**Fig 3:**
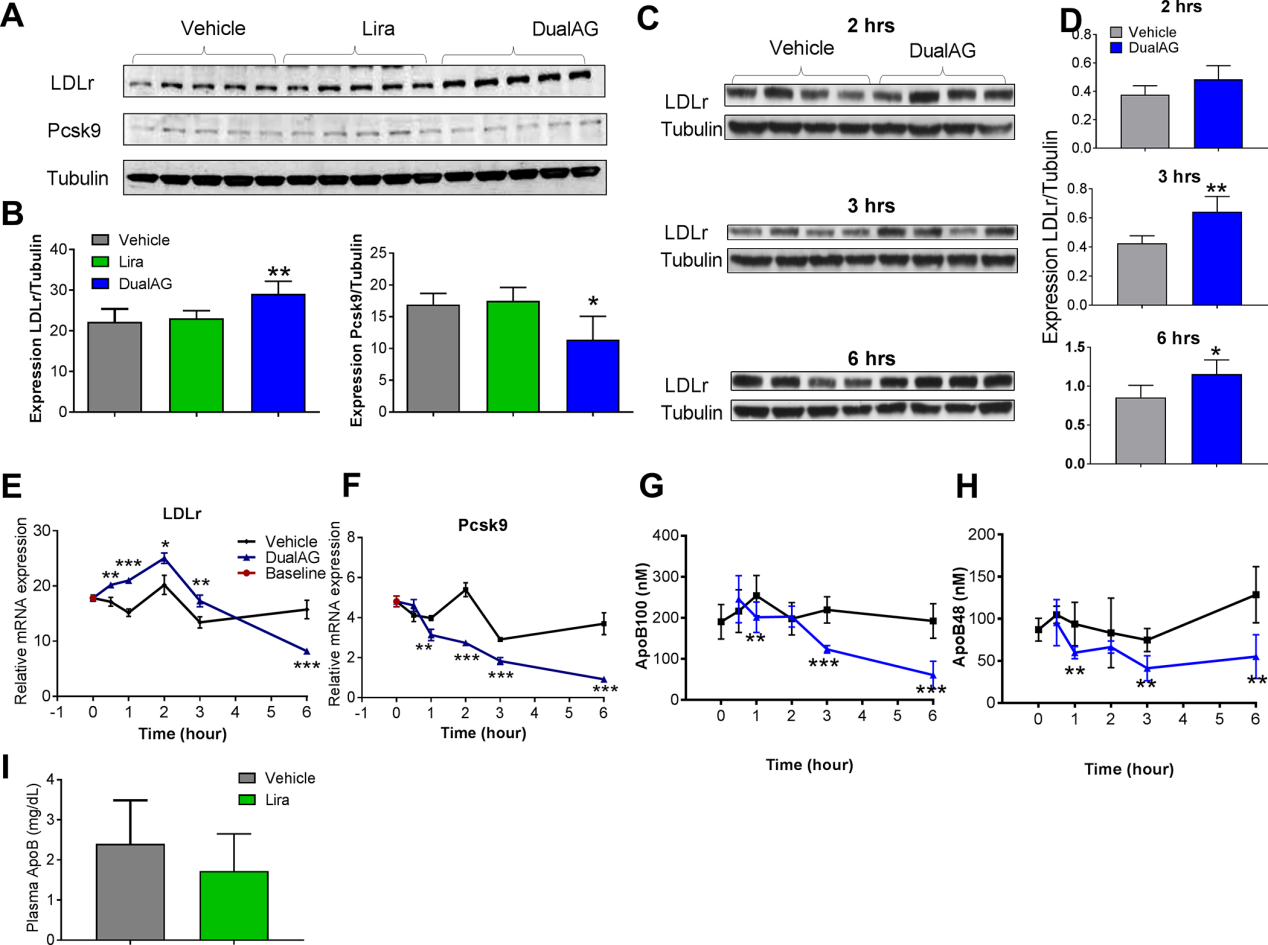
DualAG induced elevation of LDL receptor (LDLr) expression in livers of DIO mice. Mice were fasted for 2hrs followed by injection of vehicle, Liraglutide (25nmol/kg) or DualAG (25nmol/kg). **A.** Hepatic protein expression of LDLr and Pcsk9 after 6 hrs of treatment injection, as determined by western blots. **B.** Blot intensity for LDLr and Pcsk9 was quantified by ImageJ and plotted after normalizing to tubulin expression. **C.** Hepatic LDLr protein expression after 2, 3, and 6 hrs of treatment injection. **D.** Blot quantification for LDLr at 2, 3, and 6 hrs after treatment injection. **E.** Relative mRNA expression of LDLr in liver. **F.** Relative mRNA expression of Pcsk9 in liver. **G.** Plasma apolipoprotein (Apo) B100 levels over the course of 6 hrs after injection of DualAG. **H.** Plasma ApoB48 levels over the course of 6 hrs after injection of DualAG]. I. Plasma levels of total ApoB in vehicle and Liraglutide treated groups at 6 hrs after injection.

The primary form of cholesterol/ TG uptake in the liver occurs through LDL-receptor (LDLr). As seen in western blots, and plotted blot intensity, hepatic LDLr protein levels were significantly elevated in response to DualAG, but not liraglutide (Fig 3A and 3B). This elevation in LDLr was accompanied by an decline in Pcsk9 protein expression. Considering the role of Pcsk9 in chaperoning LDLr for degradation, the DualAG induced decline in Pcsk9 expression is in agreement with elevated LDLr expression. Interestingly, DualAG induced elevation in LDLr protein expression was evident as early as 2 hrs after treatment. The differences in blot intensity between vehicle and DualAG were significant at 3 and 6 hrs, whereas a trend was visible at 2hrs (Fig 3C and 3D). Messenger RNA expression of LDLr increased at 0.5, 1, 2 and 3 hrs in DualAG treated group. In contrast to protein expression, mRNA expression at 6hrs was significantly down-regulated with DualAG (Fig 3E). Pcsk9 mRNA expression was significantly decreased with DualAG at 1, 2, 3 and 6 hrs post-dosing (Fig 3F). Apolipoprotein (Apo) B100 is a component of LDL, and serves as an indicator of LDL levels in plasma [14]. DualAG treatment significantly decreased ApoB levels in plasma, and the decrease was evident at 1, 3, and 6 hrs post-dosing (Fig 3G). Similarly, ApoB48 is a chylomicron and LDL associated protein [15], which was also decreased significantly at 1, 3 and 6 hrs after DualAG treatment (Fig 3H). Liraglutide treatment did not cause a significant decrease in total ApoB plasma levels after 6hrs of injection (Fig 3I).

### DualAG decreased hepatic de novo lipogenesis

**Fig 4:**
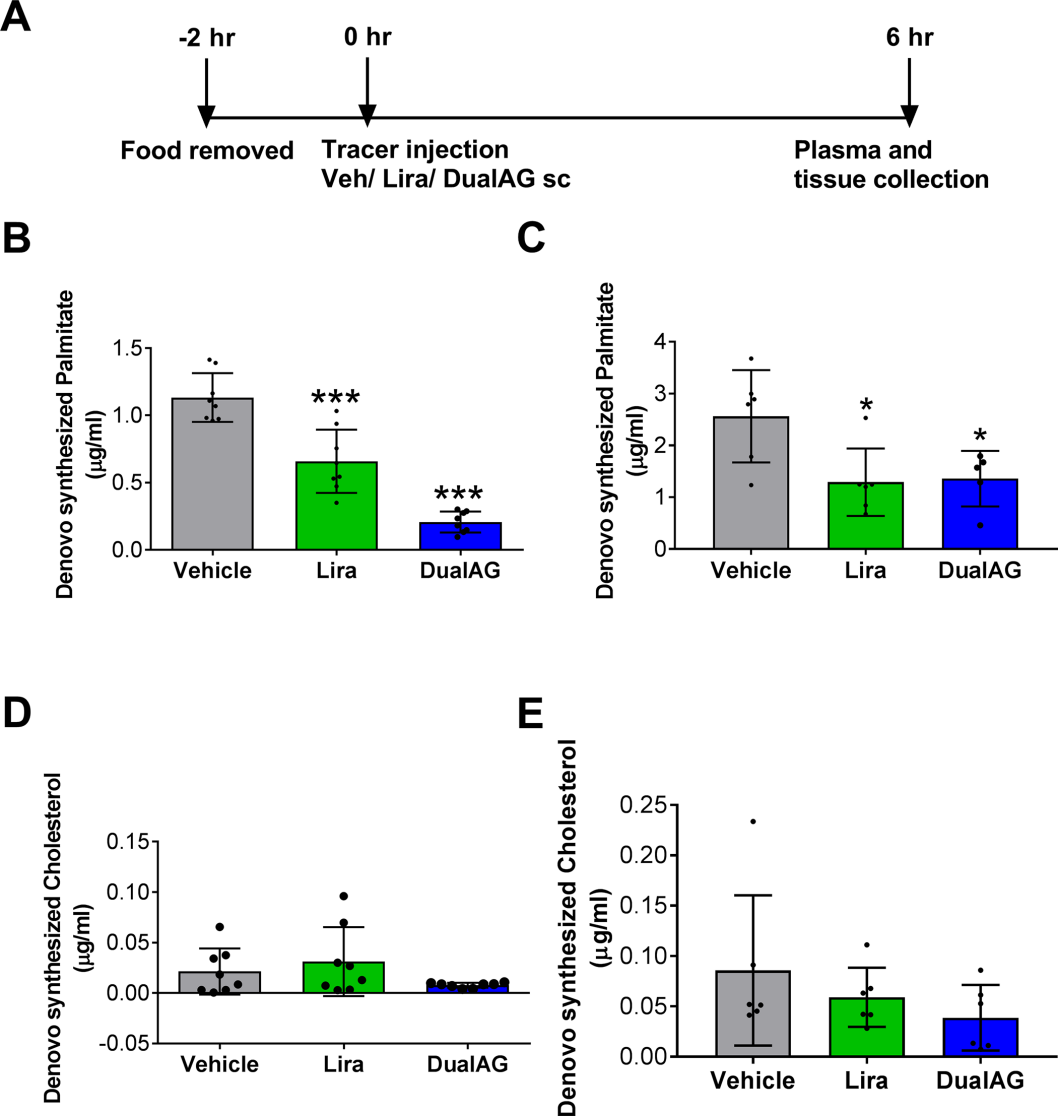
DualAG reduced *de novo* lipogenesis in DIO mice. **A.** Timeline for *de novo* lipogenesis experiments. After fasting for 2 hrs, mice were injected with 20ml/kg i.p. deuterated water (D_2_O), simultaneously with s.c. injection of vehicle, Liraglutide (25nmol/kg) or DualAG (25nmol/kg). After 6 hrs, the plasma and tissues were collected for tracer analysis. **B.** *De novo* palmitate synthesis as determined from plasma fraction. **C.** *De novo* palmitate synthesis as determined from liver tissue. **D.** *De novo* synthesized cholesterol in plasma. **E.** *De novo* synthesized cholesterol in liver tissue.

Using deuterium label tracer, newly synthesized palmitate and cholesterol was tracked under the influence of vehicle, liraglutide or DualAG treatment. As depicted in Fig 4A, D_2_O tracer injection was given 6 hrs prior to plasma and tissue collection, so the de-novo lipogenesis values obtained reflect newly synthesized palmitate/ cholesterol 6hrs. Liraglutide treatment suppressed palmitate synthesis by ~50%, whereas DualAG suppressed palmitate synthesis by more than 80% as compared to vehicle (Fig 4B). Concurrent to plasma levels of newly made palmitate, levels in liver tissue were also suppressed by both liraglutide and DualAG (Fig 4C). It was noted that levels of *de novo* synthesized cholesterol levels remained unchanged in both liraglutide and DualAG treated groups when checked in plasma as well as liver tissue (Fig 4D and 4E).

**Fig 5:**
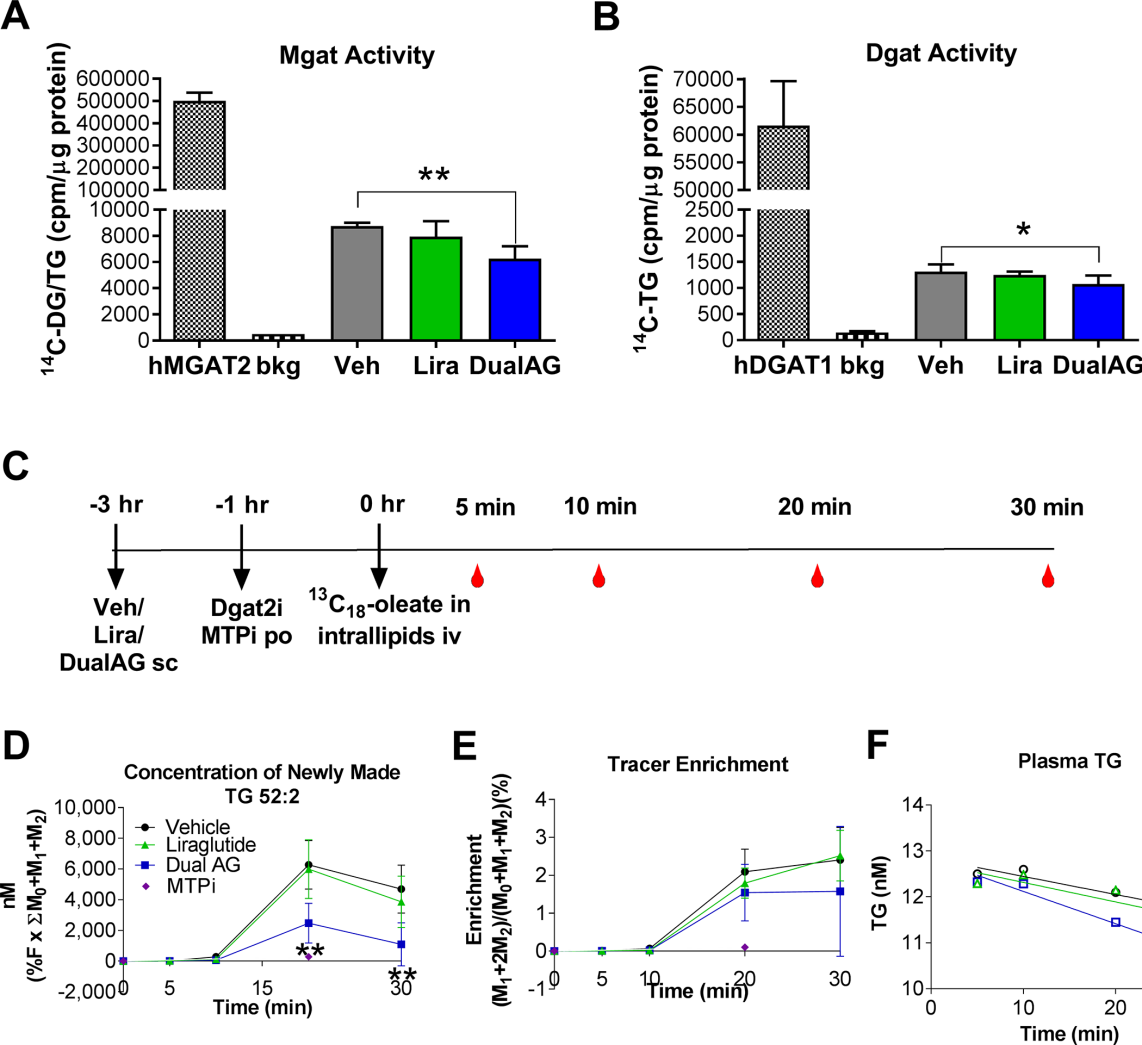
DualAG decreased TG synthesis in DIO mice. **A.** Monoacylglycerol acyltransferase (Mgat) enzyme activity. Recombinant human Mgat2 was used as a positive control, and ratio of C^14^-diacylglycerol to TG was normalized to protein content from the livers of DIO mice treated with vehicle, Liraglutide (25nmol/kg) or DualAG (25nmol/kg). **B.** Diacylglycerol acyltransferase (Dgat) enzyme activity. Recombinant human Dgat1 was used as a positive control, and amount of C^14^-TG was normalized to protein content from the livers of DIO mice treated with vehicle, Liraglutide (25nmol/kg) or DualAG (25nmol/kg). **C.** Timeline for *de novo* TG synthesis and dynamic TG metabolism experiments. DIO mice were injected with vehicle, Recombinant human Mgat2 was used as a positive control, and ratio of C^14^-diacylglycerol to TG was normalized to protein content from the livers of DIO mice treated with vehicle, Liraglutide (25nmol/kg) or DualAG (25nmol/kg) s.c., followed by oral administration of Dgat2 and Mtp inhibitors. After one hour, mice were injected with intravenous ^13^C_18_-oleate in intralipids, which was followed by blood collection at 5, 10, 20 and 30 mins (n = 4/ time point). **D.** Plasma concentration of newly made TG that incorporated ^13^C_18_-oleate, which is an indicator of *de novo* TG synthesis and TG release from liver to blood. **E.** Overall ^13^C_18_-oleate enrichment in plasma monoacyl glycerol, diacylglycerol and TG, indicator of *de novo* synthesis. The percentage enrichment of 13C18-oleate tracer in plasma TG 52:2 was calculated as the ratio of labeled isotopologues (M18 = TG52:2 incorporating 1 equivalent of 13C18 and M36 = TG 52:2 incorporating 2 equivalents of 13C18) to total TG 52:2 (sum of all isotopologues, M0, M18 and M36). **F.** Plasma TG levels over 30 minutes of experiment, suggesting clearance of unlabeled TG.

Fatty acids are incorporated into TG through stepwise enzymatic esterification catalyzed by monoglyceride acyltransferase (Mgat) and diglyceride acyltransferase (Dgat). After treatment with DualAG, enzymatic activity of both Mgat and Dgat were significantly decreased in liver extracts of the mice. This decrease was not evident in liraglutide treated groups (Fig 5A and 5B). In order to assess dynamic TG homeostasis, we utilized ^13^C_18_-oleate tracer measurements. Intravenous administration of ^13^C_18_-oleate was followed by tracking the content in plasma, tracer enrichment and its incorporation into newly made TG. The experimental design is described in Fig 5C, which involved treatment with vehicle, Liraglutide or DualAG 3 hrs prior to ^13^C_18_-oleate administration. After 20 minutes of ^13^C_18_-oleate injection, it was demonstrated that DualAG suppressed tracer incorporation into TG by ~30% relative to vehicle (Fig 5D). This concentration of newly made TG 52:2 is a measure of fatty acid esterification into TG, as well as clearance from plasma [12]. We selected TG 52:2 as a prototypical triglyceride for measurement in our studies primarily for 2 reasons: 1) TG 52:2 is quantitatively the 2nd most abundant single molecular species contributing to the plasma triglyceride pool [16] and we reasoned that its changes would be a reasonable surrogate for “total TG” on that basis. 2) We have previously shown this analyte to respond to pharmacological perturbations affecting triglyceride metabolism, including inhibition of DGAT1 [17, 18]. Overall tracer enrichment in plasma also appeared lower in DualAG treated group; however it was not statistically significant (Fig 5E). Liraglutide treatment did not have any significant impact on new TG synthesis or tracer enrichment.

**Fig 6:**
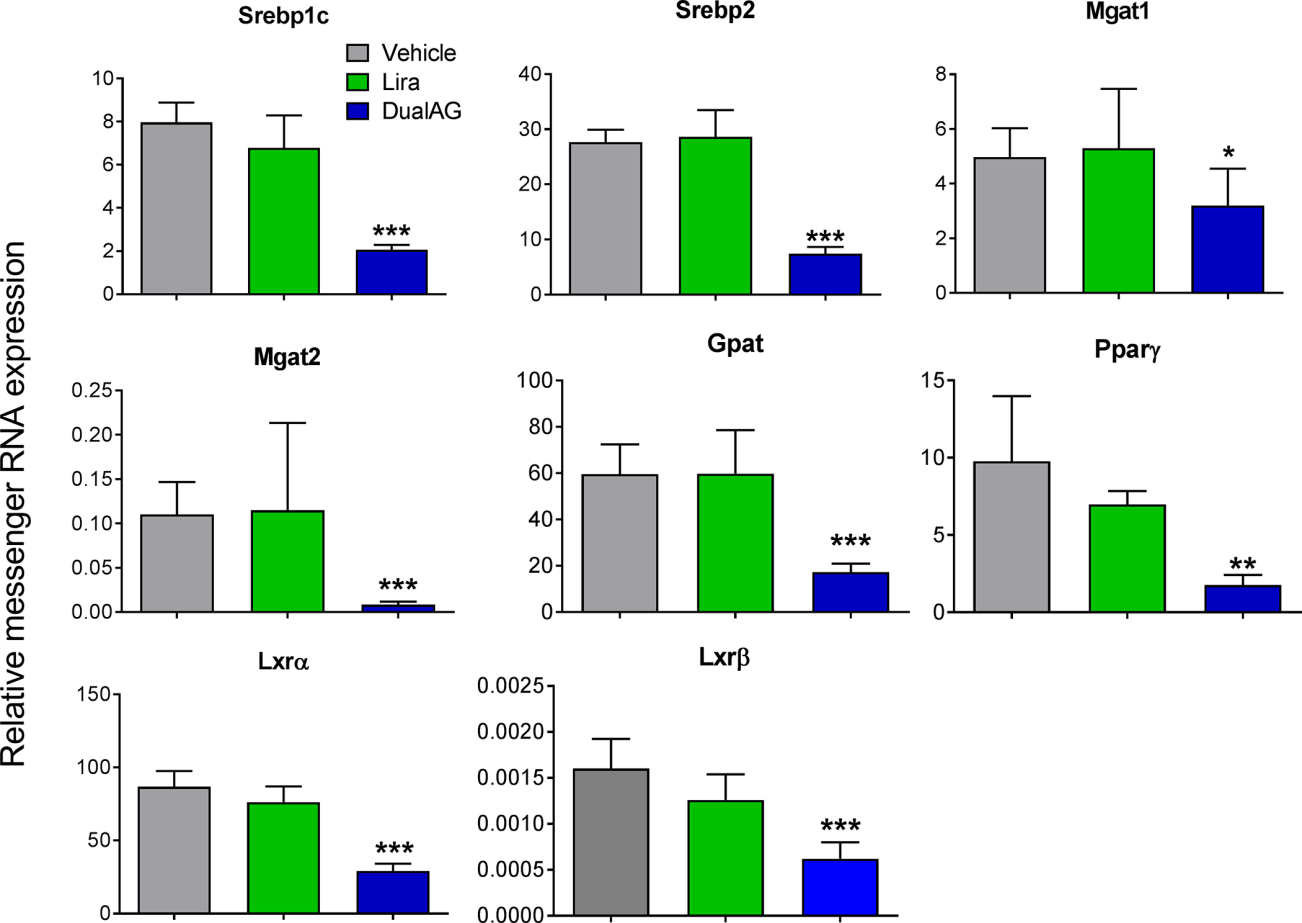
DualAG suppressed mRNA expression of key lipogenic transcription factors and enzymes in livers of DIO mice. Sterol regulatory element binding protein (Srebp) 1c, Srebp2, monoacylglycerol acyltransferase (Mgat) 1, Mgat 2, glycerol-3-phosphate acetyltransferase (Gpat), peroxisome proliferator activated receptor (Ppar) γ, liver-X-receptor (Lxr) α, and Lxrβ mRNA expression was analyzed by quantitative real-time PCR (RT-PCR).

Hepatic expression of *de novo* lipogenesis relevant targets was checked on mRNA level (Fig 6). Lipogenic regulator sterol regulatory binding protein (Srebp) 1c and Srebp2 expression was significantly suppressed by DualAG treatment, but not by liraglutide. Peroxisome proliferator activated receptor (Ppar) γ, liver X receptor (Lxr) α and β expression also showed similar changes with DualAG treatment, and remained unchanged in Liraglutide treated groups. Similar decrease in the expression of Mgat1, Mgat2, glycerol-3-phosphate acetyltransferase (Gpat) was noted for DualAG treated group.

### DualAG suppressed lipid export from liver

**Fig 7:**
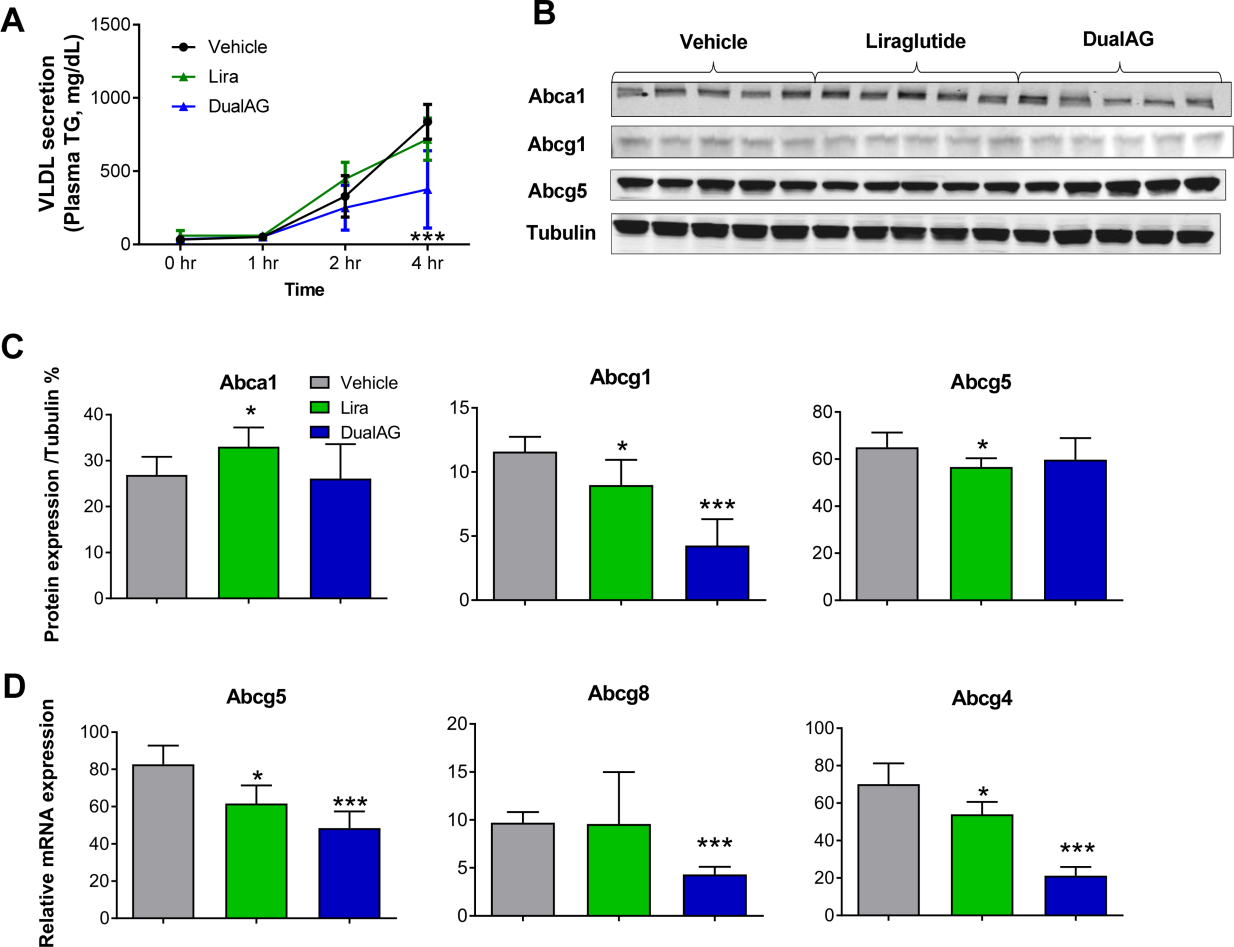
DualAG decreases VLDL secretion and associated hepatic gene/ protein expression in DIO mice. **A.** Plasma TG levels, as a measure of VLDL secretion. DIO mice were injected with poloxamer, followed by vehicle, Liraglutide or DualAG injection. The plasma was collected at 1, 2, and 4 hrs post treatment, and TG levels were determined. **B.** Hepatic protein levels of ATP-binding cassette transporters Abca1, Abcg1, and Abcg5 by western blot, after 6 hrs of treatment with vehicle, Liraglutide (25nmol/kg) or DualAG (25nmol/kg). **C.** Western blots for Abca1, Abcg1, Abcg5 were quantified and plotted after normalizing to tubulin expression. **D.** Hepatic mRNA expression of Abcg5, Abcg8 and Abcg4 determined by RT-PCR.

Nascent VLDL particles are released in order to transport TG from liver to peripheral organs. When treated with DualAG, release of VLDL from liver decreased by more than 50%. Liraglutide, on the other hand, was not able to suppress the VLDL release (Fig 7A). Protein expression of cellular lipid export relevant ATP-binding cassette transporters Abca1, Abcg1, and Abcg5 were analyzed by western blot (Fig 7B), and blot intensity quantification was plotted (Fig 7C). Abca1 expression increased with liraglutide, whereas remained unchanged with DualAG treatment. Cholesterol and phospholipid efflux transporter Abcg1 expression decreased to 80% by liraglutide, and DualAG further decreased it to 35% as compared to vehicle. Abcg5 protein expression decreased in liraglutide treatment, but remained unchanged with DualAG; on the contrary, mRNA expression of Abcg5 decreased significantly in both treatment groups (Fig 7C). Messenger RNA expression of half-transporter Abcg8, which functions as a heterodimer with Abcg5 decreased only with DualAG treatment. Abcg4 mRNA expression decreased in both liraglutide and DualAG treated groups.

### DualAG induced hepatic lipid oxidation

**Fig 8:**
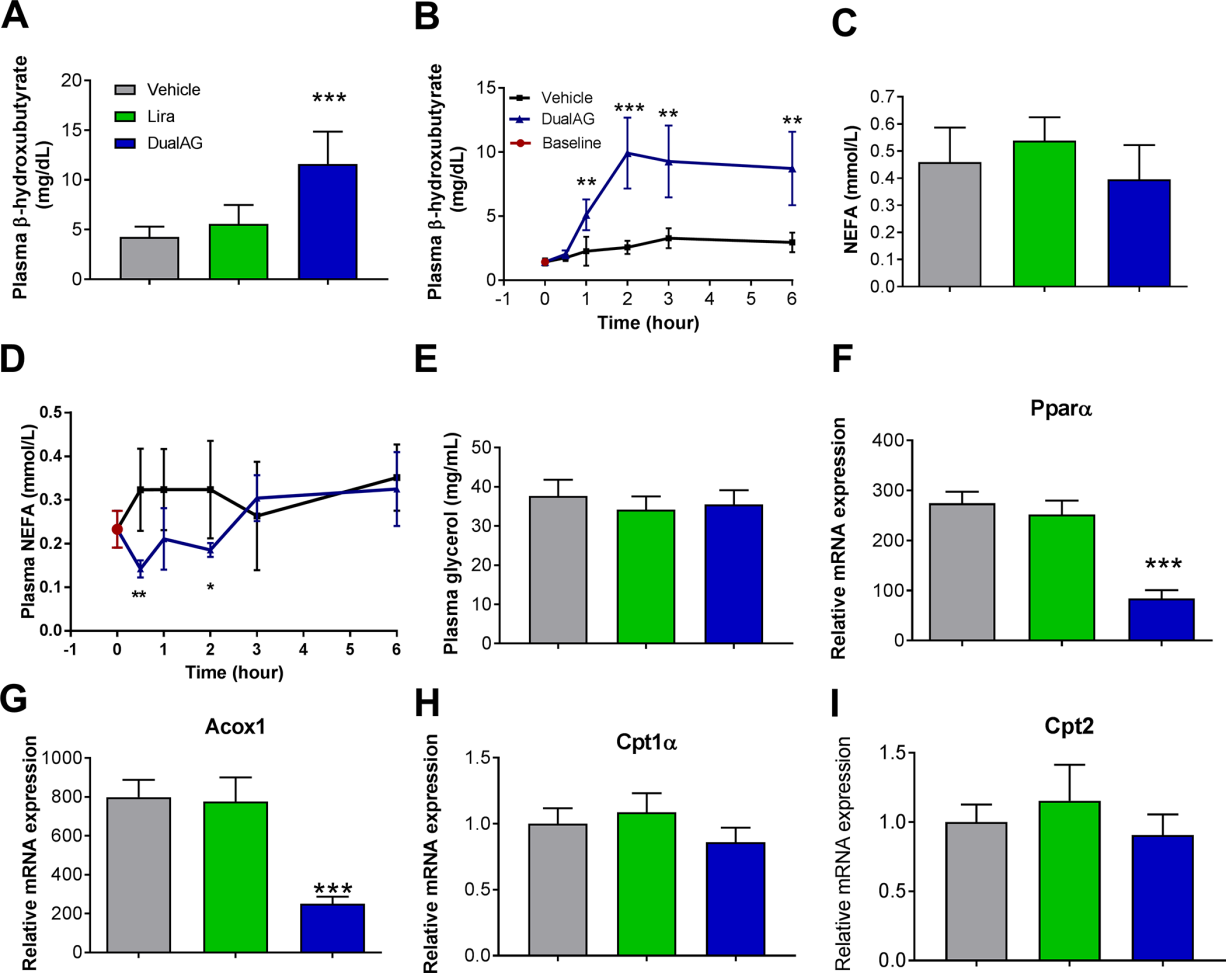
Plasma ketone bodies, non-esterified fatty acids (NEFA), glycerol and hepatic lipid oxidation target expression in DIO mice treated with DualAG. After fasting for 2 hrs, mice were injected vehicle, Liraglutide (25nmol/kg) or DualAG (25nmol/kg) subcutaneously. **A.** Plasma β-hydroxybutyrate (βHBA) levels in mice after 6 hrs of treatment injection. **B.** βHBA levels monitored over the period of 6 hrs after treatment with DualAG. **C.** Plasma NEFA levels after 6 hrs; **D.** Plasma NEFA levels from 0 to 6hrs and **E.** Plasma glycerol levels after 6 hrs of treatment. Hepatic mRNA expression of peroxisome proliferator activated receptor (Ppar) α **(F)**, acyl CoA oxidase (Acox) 1 **(G)**, carnitine palmitoyl transferase (Cpt) 1α **(H)**. Cpt2 **(I)** determined by RT-PCR.

With Gcgr activation, hepatic fatty acid oxidation was expected to be elevated in DualAG treatment. Plasma levels of the ketone, β-hydroxy butyrate (βHBA), were increased more than 2-fold in DualAG treated mice. This change was not noted in the liraglutide group (Fig 8A). DualAG induced elevation in βHBA was noted starting from 1hr post treatment and the changes remained consistent for the entire duration of the experiment (6hrs, Fig 8B). As a marker of adipose tissue lipolysis, plasma non-esterified fatty acid (NEFA) and glycerol levels were checked. Neither liraglutide nor DualAG had any impact on plasma lipolysis parameters after 6 hrs of treatment (Fig 8C and 8D). Gene expression from lipid oxidation relevant targets was not in agreement with βHBA data. DualAG treatment significantly decreased Pparα and acyl-coA oxidase (Acox1) mRNA expression in liver, however no change was noted in the liraglutide treated group (Fig 8E and 8F). Pparα target genes, carnitine palmitoyl transferase (Cpt) 1α and Cpt2 mRNA, expression remained unchanged with both liraglutide and DualAG treatments (Fig 8G and 8H).

## Discussion

In this comprehensive lipid homeostasis analysis of acute administration of Glp1/Gcgr dual agonist (DualAG), we demonstrate marked reduction in plasma TG levels in diet-induced obese (DIO) mice. Within 6 hrs of single dose treatment, DualAG reduced plasma LDL levels significantly, an effect not observed with liraglutide treatment. Moreover, the DualAG-induced reduction in plasma LDL was accompanied by increase in hepatic levels of total cholesterol. Herein, we report for the first time that acute administration of DualAG caused reduction in *de novo* fatty acid and TG synthesis, a marked increase in hepatic LDLr expression and a reduction in VLDL secretion.

In order to understand the source of the rise in hepatic cholesterol, and clearance of TG from plasma, we began by examining lipid import mechanisms in the liver. Exogenous lipids from diet are packaged in chylomicrons and make their way to the liver after transporting substantial amounts of lipids to peripheral tissues. These chylomicron remnants are picked up by hepatocytes by receptor mediated endocytosis, primarily by LDLr. The DualAG-induced decline in levels of ApoB48 further confirms enhanced chylomicron clearance from plasma. Furthermore, major lipoprotein carrying peripheral cholesterol, LDL, also enters liver through LDLr mediated endocytosis. Acute treatment with DualAG enhanced protein expression of LDLr in the liver, an effect observed as early as 3 hrs post dose. A concurrent decline in protein expression of Pcsk9 was noted, which could explain the increase in LDLr expression [19]. Levels of ApoB100, which is an apolipoprotein component of LDL, were significantly reduced in DualAG treated mouse plasma, further supporting the notion of enhanced LDL clearance by DualAG. It is evident that these changes in Pcsk9, ApoB48, ApoB100, and hepatic LDLr can be attributed to Gcgr action, as activation of Glp1 alone (Liraglutide) was not able to produce these effects.

Hepatic *de novo* lipogenesis is a major source of endogenous lipids. Synthesis of unsaturated fatty acids is followed by their saturation and/or elongation. Multiple enzyme complexes from the cytosol catalyze the transformation of precursor acetyl CoA to fatty acids. Using a tracer based approach we thoroughly analyzed the influence of DualAG and liraglutide on hepatic *de novo* lipogenesis. Deuterated water incorporation into newly synthesized palmitate was significantly declined in both liraglutide and DualAG groups; however, the extent of decrease was more prominent in DualAG. It was previously observed that silencing of glucagon signaling caused increase in *de novo* lipogenesis as well as enhanced lipogenic gene expression [10], suggesting a role of glucagon in regulating hepatic lipids. Contrary to glucagon, insulin is known to induce the *de novo* lipogenesis, and the signaling remains intact even in an insulin resistant state [20].

Fatty acyl CoA further undergoes esterification to mono-, di- and triacylglycerols. These reactions, catalyzed by Mgat and Dgat, are essential for creating storage form of lipids, as well as for export to the periphery. Here, we demonstrate that acute DualAG administration suppressed Mgat and Dgat activity. These data were substantiated by findings from our ^13^C_18_-oleate infusion studies. Shortly after intravenous infusion of ^13^C_18_-oleate through intralipids, plasma levels of tracer enriched TG were monitored. DualAG markedly reduced newly synthesized TG in plasma, which is an indication of reduction in FFA esterification as well as TG clearance from plasma. Both Mgat, Dgat acivities, as well as FFA esterification remained unchanged with liraglutide treatment, indicating contribution of Gcgr signaling in lipid relevant effects of DualAG.

Major export of lipids from liver occurs through nascent VLDL particles rich in TGs. After release from liver, the VLDL particles acquire ApoCII and ApoE to mature. Once VLDL comes in contact with lipoprotein lipase, TGs are released and the VLDL remnant, also called intermediate density lipoprotein (IDL), travels back to the liver through the circulation. Here, we identify marked reduction in VLDL secretion from liver after treatment with DualAG. Poloxamer-triggered accumulation of TGs was measured, which is a direct indicator of VLDL secretion from liver [21]. Cholesterol and phospholipid transporters Abca1, Abcg1, Abcg5, Abcg8 were measured, and uniform pattern of change was missing. Decline in Abcg5, Abcg8 and Abcg4 mRNA expression in DualAG treated groups was accompanied by unchanged protein levels of Abca1 and Abcg5. VLDL is the precursor for formation of LDL when lipid export occurs at the periphery. The combined effects of decreased VLDL secretion, along with high LDL uptake at the liver may explain the lowering of plasma LDL and total cholesterol after DualAG treatment.

After exploring possible sources of lipid accumulation in the liver, and reasons for enhanced clearance from plasma, we performed experiments addressing hepatic lipid metabolism. Free fatty acids are metabolized in hepatocytes via enzymatic oxidation, leading to formation of acetyl CoA. Acetyl CoA formed from this process can further undergo oxidation to form water and carbon dioxide, or lead to formation of other ketone bodies including acetoacetyl CoA and β-hydroxybutyrate (βHBA). Acute treatment with DualAG significantly increased ketone (βHBA) levels in plasma. This increase was observed as early as 1 hr post dosing. Pparα is a master regulator of hepatic fatty acid oxidation, controlling transcription of carnitine palmitoyl transferase (Cpt) 1α, Cpt2, and acetyl CoA oxidase (Acox) 1 [22]. Owing to the increase in βHBA levels, we expected Pparα and target gene expression to be upregulated. Suprsingly however, significant decrease in expression of Pparα, Cpt1α, Cpt2, and Acox1 was noted in DualAG treated groups; liraglutide treatment did not change any lipid oxidation targets in liver. In the acute DualAG treatment paradigm, majority with observations in liver suggest dominant glucagon actions, as opposed to Glp1. Also, comparison with Liraglutide data shows that Gcgr agonism takes precedence in hepatic lipid regulation when it comes to acute DualAG treatment.

Overall, here we demonstrate that hepatic lipid homeostasis alterations play a vital role in DualAG improved plasma lipids. DualAG acutely decreased plasma levels of TG and cholesterol, and these changes are in agreement with decreased *de novo* lipogenesis, enhanced LDLr expression, decreased VLDL secretion and elevated fatty acid oxidation. Liraglutide also suppressed *de novo* fatty acid synthesis, but had no effect on fatty acid esterification to TGs. Also, lipid oxidation and LDLr expression remained unaffected following liraglutide treatment. These findings provide evidence of beneficial effects of Gcgr activation along with Glp-1, in addition to superior efficacy in primary endpoints (glucose and insulin). Improvement of plasma lipid profile by DualAG can be primarily attributed to hepatic effects. Also, these changes in lipid support the weight loss that has been reported previously with dual Glp1/Gcgr agonists [23].

## Abbreviations

Acox: acyl CoA oxidase
βHBA: beta hydroxyl butyrate
Cpt: carnitine palmitoyl transferase
DGAT: diglyceride acyltransferase
DIO: diet-induced obese
DualAG: Glp1r/Gcgr dual agonist
Gcgr: glucagon receptor
Glp1r: glucagon like peptide 1
Lira: Liraglutide
MGAT: monoglyceride acyltransferase
Ppar: peroxisome proliferator activated receptor
RT-PCR: real time polymerase chain reaction
